# Identification of circular RNAs and functional competing endogenous RNA networks in human proximal tubular epithelial cells treated with sodium-glucose cotransporter 2 inhibitor dapagliflozin in diabetic kidney disease

**DOI:** 10.1080/21655979.2022.2031391

**Published:** 2022-02-07

**Authors:** Yi Song, Feng Guo, Yifan Liu, Fengjuan Huang, Xunjie Fan, Lin Zhao, Guijun Qin

**Affiliations:** a Department of Endocrinology and Metabolism, The First Affiliated Hospital of Zhengzhou University, Zhengzhou, China; b Academy of Medical Sciences, Zhengzhou University, Zhengzhou, China; c Department of Hepatobiliary Surgery, The First Affiliated Hospital of Sun Yat-sen University, Guangzhou, China

**Keywords:** Dapagliflozin, circRNA, mRNA, proximal tubular epithelial cells, diabetic kidney disease, competing endogenous RNAs

## Abstract

Diabetic kidney disease (DKD) is a serious diabetes complication. Sodium-glucose cotransporter 2 inhibitors (SGLT2i) are novel anti-diabetes drugs that confer clinical renal protection. However, the molecular mechanisms involved remain unclear. Here, human proximal tubular epithelial cells (PTECs) were treated with normal glucose, high glucose, and anti-diabetes agents, including SGLT2i (dapagliflozin), metformin, and dipeptidyl peptidase-4 inhibitor (DPP-4i, vildagliptin) and microarray analysis was performed. Firstly, a total of 2,710 differentially expressed circular RNAs (circRNAs) were identified. Secondly, network pharmacology and transcriptomics analyses showed that the effects of dapagliflozin on PTECs primarily involved lipid metabolism, Rap1, and MAPK signaling pathways. Metformin mainly affected the AMPK and FOXO signaling pathways, whereas vildagliptin affected insulin secretion and the HIF-1 signaling pathway. Furthermore, circRNA-miRNA-mRNA networks, real-time reverse transcription-polymerase chain reaction (RT-PCR), and fluorescence in situ hybridization (FISH) assay revealed that the expression of hsa_circRNA_012448 was increased in PTECs treated with high glucose, whereas its expression was reversed by dapagliflozin. Finally, the hsa_circRNA_012448-hsa-miR-29b-2-5p-GSK3β pathway, involved in the oxidative stress response, was identified as an important pathway mediating the action of dapagliflozin against DKD. Overall, our study provides novel insights into the molecular mechanisms underlying the effects of dapagliflozin on DKD.

## Introduction

Diabetic kidney disease (DKD) is one of the serious microvascular complications of diabetes [1]. Considering the high incidence rate (40%) of new-onset chronic kidney disease (CKD) in patients with diabetes and the limited efficacy of renin angiotensin system inhibitors [2,3], new therapeutic strategies are urgently needed to improve the renal prognosis of patients with diabetes.

Tubular atrophy, interstitial fibrosis, and rarity of tubular capillaries are closely related to renal function decline in patients with DKD [4]. Sodium-glucose cotransporter 2 inhibitors (SGLT2i) are new oral anti-diabetes agents, that can specifically bind to SGLT2 in the proximal renal tubule, reduce glucose reabsorption, promote urinary glucose excretion, and effectively reduce blood glucose [5]. Multicenter clinical trials have demonstrated that SGLT2i considerably hinders DKD progression (EMPA-REG [6], CANVAS [7], and Declare-TIMI [8]). Previous studies have indicated that SGLT2i can protect the kidneys by regulating the tubuloglomerular feedback [9]. However, renal outcome trial (ROT) studies, including CREDENCE [10] and DAPA-CKD [11], have confirmed that canagliflozin and dapagliflozin effectively reduce the risk of end stage renal disease (ESRD) and cardiovascular death in patients with or without DKD. However, the exact molecular mechanisms remain unclear.

Circular RNAs (circRNAs) feature a stable structure formed by special loop splicing [12]. These circRNAs are highly conserved and abundantly expressed in mammalian tissues [13]. Due to the lack of terminal 5′-and 3′-ends, circRNAs exhibit higher stability and resistance against RNA exonucleases [14]. Recent studies have revealed that circRNAs regulate gene expression via acting as microRNA (miRNA) sponges [15], forming RNA-protein complexes [16], and even being transcribed into proteins [17]. Emerging studies show that circRNAs participate in extracellular matrix accumulation, proteinuria formation and renal fibrosis [18,19]. However, a limited number of studies on circRNAs have focused on DKD pathogenesis [20]. Particularly, the relationship between SGLT2i and circRNAs in DKD has not been studied.

The link between SGLT2i and renal protection occurs through various pathways, some of which have been preliminarily revealed [21–23]. In this study, to explore the detailed underlying molecular mechanisms, a comprehensive study was carried out to investigate the effects of the SGLT2i dapagliflozin on PTECs in DKD. CircRNA and mRNA microarrays were performed to systematically identify differentially expressed (DE) circRNAs and mRNAs in PTECs (HK-2 cell) treated with normal glucose (NG), high glucose (HG), dapagliflozin, metformin, and dipeptidyl peptidase-4 inhibitors (DPP-4i, vildagliptin). Importantly, combining with network pharmacology, transcriptome analyses, and bioinformatics analysis, circRNA‐mRNA co-expression networks and competing endogenous RNA (ceRNA) networks were identified, constructed, and functionally analyzed in HK-2 cells from a global perspective. Our findings provide new insights and a theoretical basis for further exploration of the molecular mechanisms underlying the protective action of dapagliflozin in DKD.

## Material and methods

### Cell culture and treatment

Human proximal tubular epithelial cells (HK-2 cells) were purchased from the Shanghai Chinese Academy of Sciences (Shanghai, China) and cultured in DMEM (Hyclone Laboratories, Inc., Logan, UT, USA) supplemented with 10% fetal bovine serum (Biological Industries, Beit HaEmek, Israel), 100 U/ mL penicillin, and 100 μg/ mL streptomycin at 37°C and 5% CO2 according to the standard guidelines. After synchronization by culturing in DMEM for 24 h, the cells were treated with normal glucose (5.6 mM) or high glucose (30.0 mM) for 48 h, and then treated with dapagliflozin (2.5 μM, HY-10450, MedChemExpress, MCE, USA,), metformin (5 mM, Cat. NO S1950, Selleck Chemical LLC, Huston, USA), and vildagliptin (150 μM, LAF-237, Selleck Chemical LLC, USA) for 48 h in 6-well plates (Corning Inc., Corning, NY, USA).

### Cell transfection

To overexpress hsa_circRNA_012448, the EPS15 sequences were inserted in the pcDNA3.1 vectors (GenePharma, Shanghai), whereas the mock vector lacking the hsa_circRNA_012448 sequence was used as a control. According to the manufacturer’s instructions, cell transfections were performed using lipofectamine 2000 (Invitrogen, Carlsbad, CA, USA).

### Fluorescence in situ hybridization (FISH) assay

Cy3-labeled hsa_circRNA_012448 (5'- TTGGCCTTATCTTCAGGAACTGCAAACTCATCTCT −3') (Servicebio, Wuhan, China) was used to observe the localization of hsa_circRNA_012448 in HK-2 cells. A fluorescence in situ hybridization (FISH) assay was performed using a Fluorescent In Situ Hybridization Kit (GenePharma, Shanghai) according to the manufacturer’s instructions. Cell nuclei were stained with 4,6-diamidino-2-phenylindole (DAPI, Beyotime, China). Images were obtained using a fluorescence microscope (Olympus, Japan).

### Measurement of reactive oxygen species (ROS) genetation

The intracellular production of ROS was detected using the 2'7-dichlorodihydrofluorescein-diacetate (DCFH-DA) probe (Beyotime, Shanghai, China) by fluorescence microscope according to the manufacturer’s instructions. Briefly, after treatments for the indicated time, cells were washed three times with serum free medium, and incubated with DCFH-DA at a final concentration of 10 mmol/L in serum free medium at 37°C for 40 min, the washed twice and analyzed by fluorescence microscope (Olympus, Japan).

### CircRNA and mRNA microarrays

Three pairs of HK-2 cells from each group (NG, HG, dapagliflozin, metformin, and vildagliptin) were used for circRNA and mRNA microarray analysis by KangChen Biotech Inc (Shanghai, China). Sample labeling and microarray hybridization were conducted using the Arraystar Human circRNA Array v2.0 (Arraystar). Briefly, total RNA was digested with RNase R (Epicenter, Madison, WI, USA). Then, linear RNAs were removed and circular RNAs were concentrated. Subsequently, the concentrated circRNAs were amplified and transcribed into fluorescent RNAs using the Arraystar Super RNA Labeling Kit and the random priming method. Thereafter, the labeled cRNAs were hybridized, washed, and scanned on Arraystar Human circRNA Array v2.0 (Arraystar), using an Agilent Scanner G2505C (Agilent Technologies, Santa Clara, CA, USA). Differentially expressed circRNAs were identified based on a *P*-value of < 0.05 and a fold change of > 1.0. For mRNAs, we used Arraystar Human mRNA arrays (Arraystar) following the manufacturer’s instructions and quantile standardization was performed on the original data using the GeneSpring GX v12.1 software (Agilent Technologies). Differentially expressed mRNAs were identified based on a *P*-value of < 0.05 and a fold change of > 2.0. All raw data were deposited in the Gene Expression Omnibus under the accession code GSE179226 and GSE158534.

*RNA isolation and validation using real-time reverse transcription-polymerase chain reaction (RT-PCR)*

Total RNA was isolated from HK-2 cells using TRIzol (Takara Bio Inc, Japan). The RNA concentration was assessed using a NanoDrop ND-1000 spectrophotometer (Thermo Scientific, Wilmington, DE, USA). Total RNA was reverse transcribed to complementary DNA using a reverse transcription kit (Sangon Biotech, Shanghai, China). For circRNAs, total RNA was digested with RNase R (Epicenter, Madison, WI, USA) according to the manufacturer’s instructions. Then, the expression of circRNAs was assessed using RT-PCR and the SGExcel FastSYBR Mixture (with ROX) (Sangon Biotech) in an ABI 7500 system (Applied Biosystems, CA, USA). Ten predicted miRNAs were reverse transcribed into cDNA using an miRNA First Strand cDNA Synthesis kit (Tailing Reaction) (Sangon Biotech). The expression of miRNAs was assessed using RT-PCR and a MicroRNA qPCR Kit (Sangon Biotech). GAPDH, β-actin and U6 served as the internal normalization controls. Target gene relative fold expression was determined using the 2^−ΔΔCt^ method. Primer sequences are listed in Table 1.

**Table 1.** Primers used for RT-PCR

Genes	Forward	Reverse
hsa_circ_001586	GAGAGTCACCACAAGGCCAA	GTAACGGTGGGGCTTCTTCA
hsa_circ_102129	GCCAGACTTCAGGGTAAGCC	CCCCAGCTGAAACCTTCCAT
hsa_circRNA_102230	AGTCAACGACAAGAACCCACA	GTCGCCTTGTCCTCCACCTT
hsa_circRNA_103933	AAGGCATACGTCATCGGACC	GAAACAGGAGCACCTTAGACCA
hsa_circ_012448	TGGGAGTTGAGTGATATTGACCATG	CATTCACTGGGCTTAAACTATCAAA
hsa_circRNA_092556	TGGCGGCGCAACTCCGAAGAAGA	CTGCCCCAACTGGCTTCTTAGGTTT
hsa_circRNA_400025	GTGCTTGCCTTTGACTCCCAATACC	GAGGGGTCCAGAACACGGGAGCCA
hsa_circRNA_055947	GGACATTAAACATGTTGGCTGT	TTACTGGTGGGTATTTCTGGG
hsa_circRNA_104979	TTAAGCAGCAGCGGTTCG	GCTTTGTTATCAGCTGGCGTA
hsa_circRNA_103046	ATGTTAGCTCAGTGCCTCTAGC	CAATAGGTTCTCTGTGATGCCT
hsa_circRNA_102092	GGTCTCTGCAAGAACACATCAT	TAGCACTCCATCAGGCCATC
hsa_circRNA_101033	TTTCATCAGGACCATCTGTTCG	TTTCATTTCCGGGTGTTCTTTC
hsa_circRNA_103667	TGCCAGATGATCAGTGGGTT	CCTGTGTTAGTTGGGGCAGA
hsa_circRNA_103664	TCCAGATCTGCGAATGTTTCT	TGCTGGGTTTATTTGGTTGCA
hsa_circRNA_034804	GGAGACATCAGTGGACACCT	CCCAGAGCATGAAATCAGCC
hsa_circRNA_100927	TGGCTTCAAGAAACACGTTGT	ACATTCATCTCATTTGTGCACTG
hsa_circRNA_004466	CCCCAGTGAAGTTGTGTGAC	GAAGTTTCCTGGGGTGCTAC
hsa_circRNA_100919	TACATGGGGAGGGCAACTAC	AGATAGACCAGTGCTTGCCA
VAV1	CAACCTGCGTGAGGTCAAC	ACCTTGCCAAAATCCTGCACA
CDKN1A	AGGTGGACCTGGAGACTCTCAG	TCCTCTTGGAGAAGATCAGCCG
IL1A	TGTATGTGACTGCCCAAGATGAAG	AGAGGAGGTTGGTCTCACTACC
HIST1H1C	ACTTGGTCTCAAGAGCCTGGTG	GGCTTCTTAGGTTTGGTTCCGC
DHRS13	CTTTGCCACTGCCTTTCTGAGC	GATGTGTCAGCAGAAAGGGACC
LAT2	GCAAGCAGAAAACCACAGAGACA	AGAGGGACAGAGACCAGAAGTG
GSK3β	CCGACTAACACCACTGGAAGCT	AGGATGGTAGCCAGAGGTGGAT
VEGFA	TTGCCTTGCTGCTCTACCTCCA	GATGGCAGTAGCTGCGCTGATA
β-actin(HUMAN)	CACCATTGGCAATGAGCGGTTC	AGGTCTTTGCGGATGTCCACGT
GAPDH(HUMAN)	GGGAAACTGTGGCGTGAT	GAGTGGGTGTCGCTGTTGA
hsa-miR-29b-2-5p	CCTGGTTTCACATGGTGGCTTAG	ACGCTTCACGAATTTGCGT
hsa-miR-378 g	CACTGGGCTTGGAGTCAGAAG	ACGCTTCACGAATTTGCGT
hsa-miR-1179	CGCAAGCATTCTTTCATTGGTTGG	ACGCTTCACGAATTTGCGT
hsa-miR-1324	CGCCAGACAGAATTCTATGCACTTTC	ACGCTTCACGAATTTGCGT
hsa-miR-6757-3p	AACACTGGCCTTGCTATCCC	ACGCTTCACGAATTTGCGT
hsa-miR-619-5p	TATATAGCTGGGATTACAGGCATGAGC	ACGCTTCACGAATTTGCGT
hsa-miR-4739	AAGGGAGGAGGAGCGGA	ACGCTTCACGAATTTGCGT
hsa-miR-7851-3p	TACCTGGGAGACTGAGGTTGG	ACGCTTCACGAATTTGCGT
hsa-miR-5787	TATATATATAGGGCTGGGGCGCG	ACGCTTCACGAATTTGCGT
hsa-miR-1273 g-3p	TATACCACTGCACTCCAGCCT	ACGCTTCACGAATTTGCGT
U6(HUMAN)	CTCGCTTCGCAGCUACA	ACGCTTCACGAATTTGCGT

### Construction of circRNA and mRNA co-expression analysis

The co-expression analysis was based on calculating the Pearson correlation coefficient (PCC) between circRNAs and mRNAs according to their expression levels [24–26]. An absolute PCC value of ≥ 0.95, *P*-value of < 0.05, and the false discovery rate (FDR) of < 0.01 were recommended and retained for further analysis.

### Construction of circRNA-miRNA-mRNA network

According to the ceRNA hypothesis, circRNAs compete for the same miRNA response element and act as molecular sponges for miRNAs, thereby regulating the de-repression of all target genes of the respective miRNA family [27]. The circRNA-miRNA networks were predicted using a home-made miRNA target prediction software from Arraystar based on TargetScan [28] (http://www. targetscan.org), miRDB [29] (http://www.mirdb.org/), and the circular RNA Interactome (CircInteractome) [30] (https://circinteractome.nia.nih.gov/) software. miRNA target gene mRNAs were downloaded using the miRTarbase [31] (http://mirtarbase.mbc.nctu.edu.tw/php/index.php) software. Finally, the regulatory networks of circRNA-miRNA-mRNA were constructed using the Cytoscape 3.7.1 software [32] (http://www.cytoscape.org/).

### Construction of protein-protein interaction (PPI) network and identification of hub genes

The PPI network of the differentially expressed genes (DEGs) was established using the STRING database with high confidence (≥ 0.90) [33] (http://string-db.org/), and then visualized using the Cytoscape 3.7.1 software (http://www.cytoscape.org/). Subsequently, the cytoHubba app in Cytoscape was used to determine the hub genes according to a previously used method [34].

### Functional enrichment analysis

To understand functional enrichment, GO and KEGG pathway analyses of DEGs were performed using the Database for Annotation, Visualization, and Integration Discovery (DAVID) [35]. The top eight GO terms and KEGG pathways were selected. A *P* value of < 0.05 was considered statistically significant.

### Analysis of network pharmacology

By screening the STITCH database [36] (http://stitch.embl.de/), the targets for dapagliflozin, metformin, and vildagliptin were selected. Using the DisGeNET database [37] (http://www.disgenet.org/), the DKD targets were predicted. Then using the Venny analyses, the common target genes were selected from these two databases. The top eight KEGG pathways were selected and considered statistically significant at a *P* value of < 0.05.

### Statistical analysis

Comparisons between two groups were analyzed using the Student’s t-test, and multiple comparisons were calculated using one-way analysis of variance (ANOVA) followed by Bonferroni’s multiple comparisons test using the GraphPad Prism software (version 9.0). Data are presented as the mean ± S.D. A *P*-value of < 0.05 was considered statistically significant.

## Results

The possible protection mechanisms of SGLT2i against DKD have not been comprehensively elucidated. Here, we first used network pharmacology approach and transcriptome microarray analysis to identify the candidate targets and potential pathways of dapagliflozin, metformin, and vildagliptin in DKD in HK-2 cells. Then, we identified the key circRNAs, hub genes, and pathways that were highly related to the effects of dapagliflozin on HK-2 cells by performing an integrated bioinformatic analysis, including differential expression analysis, GO and KEGG enrichment analyses, PPI network analysis, CNC network analysis, ceRNA network analysis, and qRT-PCR analysis. Finally, we identified hsa_circRNA_012448-hsa-miR-29b-2-5p-GSK3β as an important pathway for dapagliflozin against DKD by performing functional assays. This study provides novel insight into the molecular mechanisms underlying the action of dapagliflozin in DKD.

### Network pharmacology analysis of dapagliflozin, metformin and vildagliptin treatment of DKD

**Figure 1.**
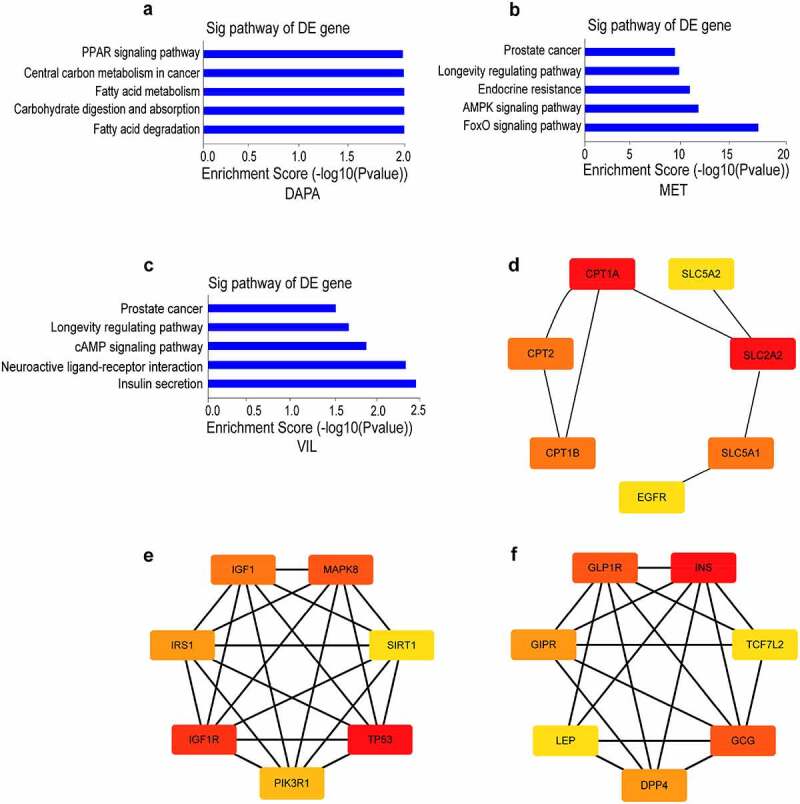
**Network pharmacology analysis of dapagliflozin, metformin and vildagliptin treatment of DKD** (a)-(c) KEGG pathway analysis for the predicted target genes of different drugs on DKD using DisGeNET database and Venny analysis (**a**. dapagliflozin, **b**. metformin, **c**. vildagliptin). (d)-(f) Identification of hub genes for the predicted target genes of different drugs on DKD using cytoscape 3.7.1 software (**d**. dapagliflozin, **e**. metformin, **f**. vildagliptin). Color shade represent the degree of correlation. DAPA: dapagliflozin, MET: metformin, VIL: vildagliptin.

Using network pharmacology analysis, we predicted a total of 47, 37, and 18 targets for dapagliflozin, metformin, and vildagliptin on STITCH [36] (http://stitch.embl.de/), respectively. KEGG pathway enrichment analyses showed that the targets for dapagliflozin were enriched in fatty acid degradation and metabolism, PPAR, adipocytokine, and peroxisome signaling pathways. The targets for metformin were mainly enriched in FOXO, AMPK, insulin, and PI3K-Akt signaling pathways, whereas those for vildagliptin were enriched in insulin secretion, cAMP, and maturity onset diabetes of the young pathways (Figure S1). Then we predicted 1,189 targets of DKD using the DisGeNET [38] (https://www.disgenet.org/) database, and obtained 7, 19, and 9 targets for dapagliflozin, metformin, and vildagliptin using Venny (http://bioinfogp.cnb.csic.es/tools/venny/index.html) analysis, respectively. Using KEGG pathway analysis, the targets of dapagliflozin involved in protecting DKD distributed in the fatty acid metabolism and degradation, carbohydrate digestion and absorption, and the PPAR signaling pathway. The targets of metformin involved in protecting DKD distributed in the FOXO, AMPK, and longevity regulating signaling pathways, while those of vildagliptin were enriched in neuroactive ligand-receptor interaction, the cAMP signaling pathway, and insulin secretion (Figure 1a-c). In addition, we obtained the hub genes of different drugs involved in protecting DKD using the cytoHubba app in Cytoscape. In these networks, SLC2A2, IGF1R, and INS were the core genes mediating the effects of dapagliflozin, metformin, and vildagliptin on DKD, respectively (Figure 1d-f).

### CircRNA and mRNA expression profiles in HK-2 cell treated with dapagliflozin, metformin, or vildagliptin

**Figure 2.**
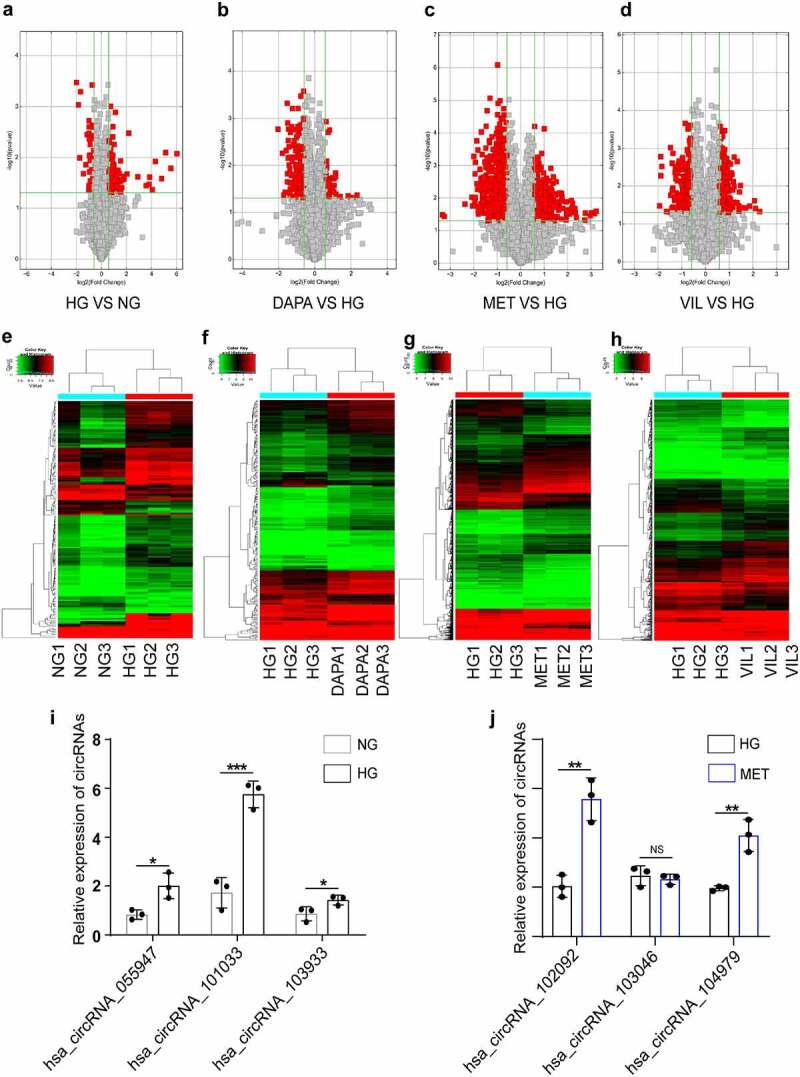
**CircRNA and mRNA expression profiles in HK-2 cell treated with dapagliflozin, metformin, or vildagliptin** (a)-(d) The volcano plot of differentially expressed circRNAs (**a**. HG vs NG, **b**. dapagliflozin vs HG, **c**. metformin vs HG, **d**. vildagliptin vs HG) (Fold-change > 1.0 and *P*-value < 0.05). (e)-(h) Cluster analysis of differentially expressed circRNAs (**e**. HG vs NG, **f**. dapagliflozin vs HG, **g**. metformin vs HG, **h**. vildagliptin vs HG) (Fold-change > 1.0 and *P*-value < 0.05). (i)-(j) Validation of randomly selected circRNAs using RT-PCR. *P < 0.05, **P < 0.01, ***P < 0.001, NS: not significant. NG: normal glucose, HG: high glucose, DAPA: dapagliflozin, MET: metformin, VIL: vildagliptin.

To clarify the precise mechanisms of different drugs on DKD, we conducted RNA microarrays to explore the role of differentially expressed circRNAs and mRNAs in HK-2 cells treated with NG, HG, dapagliflozin, metformin, and vildagliptin. A total of 2,710 significantly DEcircRNAs were identified between the five groups (fold change ≥ 1.5, *P* < 0.05), whereas 119 upregulated circRNAs and 434 downregulated circRNAs were identified between the NG and HG group. Compared with the HG group, a total of 1,406 upregulated circRNAs and 1,151 downregulated circRNAs were identified in the drug groups (185 upregulated and 49 downregulated circRNAs in the dapagliflozin group, 901 upregulated and 736 downregulated circRNAs in the metformin group, and 320 upregulated and 366 downregulated circRNAs in the vildagliptin group) (Figure 2a-d). The hierarchical clustering heatmap displayed the characteristic DEcircRNAs in the different groups (Figure 2e-h). To validate the microarray analysis results, six circRNAs were randomly selected and the results of RT-PCR showed that five of these showed changes consistent with those of microarray analysis, including three upregulated circRNAs in the HG compared with the NG group, and two upregulated circRNAs in the metformin compared with the HG group (Figure 2i and j). Overall, the RT-PCR results supported the reliability of the microarray data, for the most part.

### Functional analyses of the parental linear transcripts for differentially expressed circRNAs

**Figure 3.**
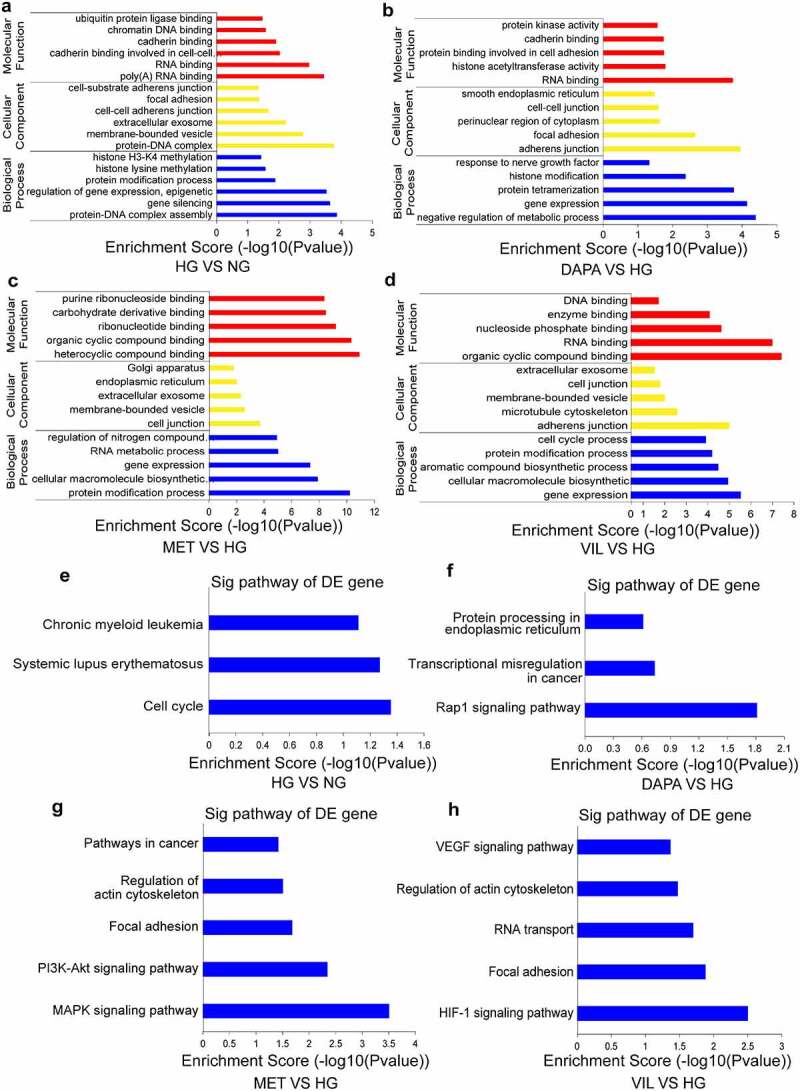
**Functional analyses of the parental linear transcripts for differentially expressed circRNAs** (a)-(d) GO annotation of the parental linear transcripts for differentially expressed circRNAs (**a**. HG vs NG, **b**. dapagliflozin vs HG, **c**. metformin vs HG, **d**. vildagliptin vs HG). (e)-(h) KEGG pathway analysis of the parental linear transcripts of differentially expressed circRNAs (**e**. HG vs NG, **f**. dapagliflozin vs HG, **g**. metformin vs HG, **h**. vildagliptin vs HG). NG: normal glucose, HG: high glucose, DAPA: dapagliflozin, MET: metformin, VIL: vildagliptin.

To provide a comprehensive understanding of the functions of circRNAs, the parental linear transcripts for circRNAs were annotated and evaluated using GO and KEGG pathway analyses. The results showed that the DEcircRNAs in the HG group were enriched into five GO biological process terms, such as protein-DNA complex assembly, gene expression and methylation (Figure 3a). DEcircRNAs in the dapagliflozin group were mainly involved in the regulation of metabolic process, gene expression and histone modification (Figure 3b), whereas protein modification processes and gene expression were enriched in the metformin and vildagliptin groups, respectively (Figure 3c and d). KEGG pathway analyses showed that the DEcircRNAs in the HG group were mainly involved in the cell cycle (Figure 3e). However, in the dapagliflozin group, the role of DEcircRNAs was closely related to the Rap1 signaling pathway, transcriptional misregulation in cancer, and protein processing in the endoplasmic reticulum (Figure 3f), whereas metformin was associated with the MAPK and PI3K-Akt signaling pathways (Figure 3g), and vildagliptin participated in the HIF-1 signaling pathway, focal adhesion and VEGF signaling pathways (Figure 3h). Collectively, these biological processes and pathways may guide further exploration of the molecular mechanisms underlying the effects of different drugs on DKD.

### Functional analyses of genes co-regulated by dapagliflozin, metformin, and vildagliptin

**Figure 4.**
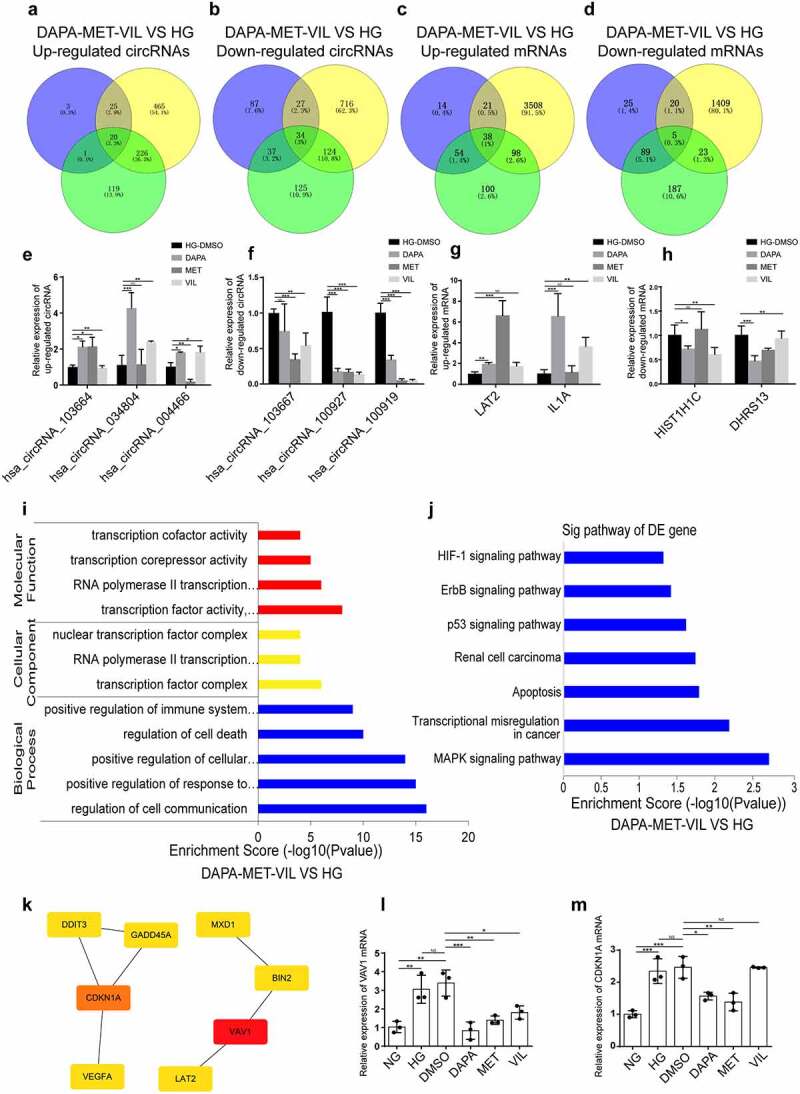
**Functional analyses of genes co-regulated by dapagliflozin, metformin, and vildagliptin** (a)-(b) Venny diagram analysis of differentially expressed circRNAs co-regulated by dapagliflozin, metformin, and vildagliptin. (c)-(d) Venny diagram analysis of differentially expressed mRNAs co-regulated by dapagliflozin, metformin and vildagliptin. (e)-(f) Validation of the up-regulated and down-regulated circRNAs in the HG group compared with the dapagliflozin, metformin and vildagliptin groups using RT-PCR. (g)-(h) Validation of the up-regulated and down-regulated mRNAs in the HG group compared with the dapagliflozin, metformin and vildagliptin groups using RT-PCR. (i)-(j) GO (**i**) and KEGG pathway (**j**) analysis for differentially expressed mRNAs co-regulated by dapagliflozin, metformin and vildagliptin. (k) Identification of the hub genes of differentially expressed mRNAs co-regulated by dapagliflozin, metformin and vildagliptin with cytoHubba in Cytoscape 3.7.1 software. (l)-(m) Validation of the hub genes of differentially expressed mRNAs co-regulated by dapagliflozin, metformin and vildagliptin using RT-PCR. NG: normal glucose, HG: high glucose, DAPA: dapagliflozin, MET: metformin, VIL: vildagliptin. Color shade represent the degree of correlation.

To explore if shared mechanisms underlying the actions of dapagliflozin, metformin, and vildagliptin in improving the HK-2 cell injuries induced by HG, we used Venny analysis to reveal the circRNAs and mRNAs co-regulated by the three types of drugs. The results showed that dapagliflozin, metformin, and vildagliptin jointly regulated 54 circRNAs and 43 mRNAs, with 20 upregulated circRNAs and 38 upregulated mRNAs, and 34 downregulated circRNAs and 5 downregulated mRNAs (Figure 4a-d), some of which were validated using RT-PCR (Figure 4e-h). Furthermore, GO and KEGG analyses were performed on these genes. Based on the GO terms, the biological processes, cellular components, and molecular functions of these genes distributed were mainly cell communication, transcription factor complex, and transcription factor activity (Figure 4i). Meanwhile, the significantly enriched KEGG pathways were mainly the MAPK signaling pathway, transcriptional misregulation in cancer, apoptosis, p53, ErbB, and HIF-1 signaling pathways (Figure 4j). Furthermore, the hub genes of these key pathways, VAV1 and CDKN1A (Figure 4k), were validated using RT-PCR (Figure 4l and m).

## Exploration of the relationship between the key circRNAs and their target genes related to dapagliflozin

**Figure 5.**
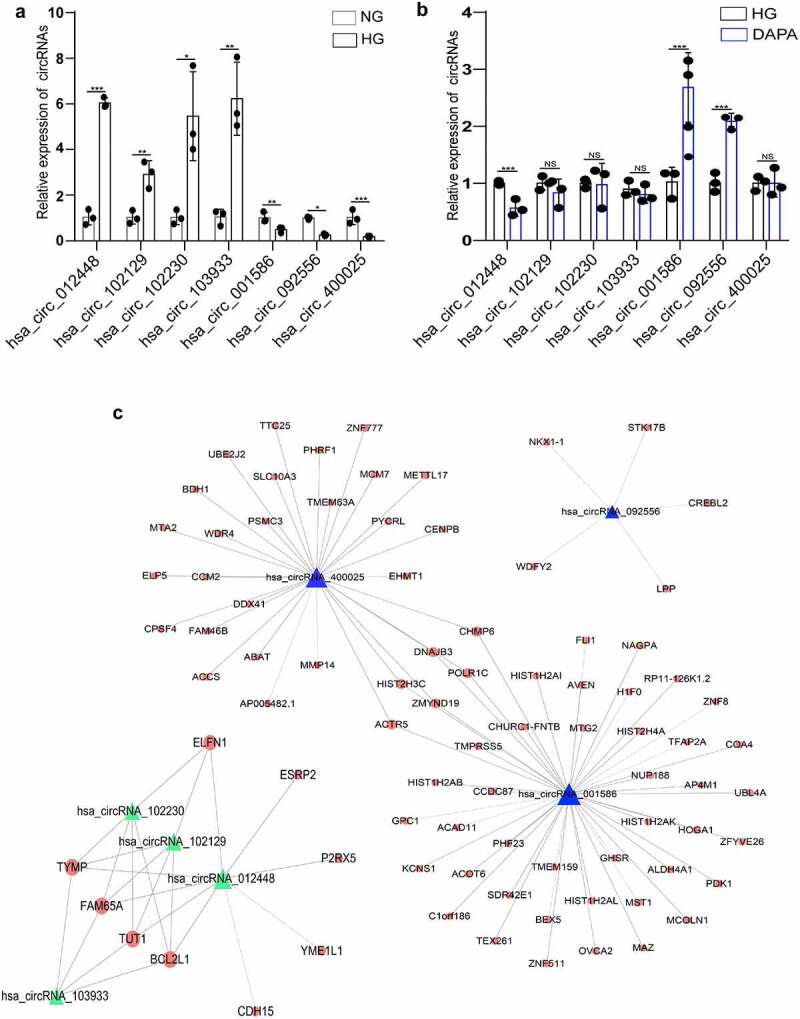
**Exploration of the relationship between the key circRNAs and their target genes related to dapagliflozin** (a) Validation of the dysregulated circRNAs in the HG group compared with the NG group. (b) Validation of the dysregulated circRNAs in the dapagliflozin group compared with the HG group. (c) The circRNA-mRNA interaction network of differentially expressed circRNAs in the NG, HG, and dapagliflozin group. *P < 0.05, **P < 0.01, ***P < 0.001. The triangle nodes represent differentially expressed circRNAs. The round nodes represent circRNA related genes. Solid lines represent positive correlation, dotted lines represent negative correlation. NG: normal glucose, HG: high glucose, DAPA: dapagliflozin.

Next, we focused on dapagliflozin related circRNAs and mRNAs. Compared with the NG group, seven significantly DEcircRNAs, including the top four upregulated circRNAs and the top three downregulated circRNAs were selected in the HG group (Table 2). These circRNAs were validated using RT-PCR and the results showed a similar trend to that of the microarray data (Figure 5a). Notably, among these candidate circRNAs, there was one circRNA that was induced by HG but repressed by dapagliflozin and two circRNAs that were repressed by HG but induced by dapagliflozin, indicating that dapagliflozin might protect DKD by regulating these circRNAs (Figure 5b). To identify the functions of these circRNAs, we constructed coding and noncoding co-expression (CNC) networks according to the correlation degree of the circRNA target genes (Figure 5c). The functional analyses showed that the target genes of upregulated circRNAs in the HG group were mainly enriched in the regulation of the endoplasmic reticulum, calcium ion transport, cell apoptosis, pyrimidine metabolism, mitophagy pathway, p53, and EGFR signaling pathways (Fig. S2a and b), whereas the target genes of downregulated circRNAs in the HG group were mainly enriched in gene expression, oxoacid metabolic process, amino acid metabolism, and lysosome pathways (Fig. S2c and d). However, the target genes of dapagliflozin-related circRNAs primarily focused on gene expression, glucose metabolic process, extracellular exosome, epigenetics, glyoxylate and dicarboxylate metabolism, amino acid metabolism, and lysosome pathways (Fig. S2e and f). These data provide a theoretical basis for further exploring the mechanistic actions of dapagliflozin.

**Table 2.** Top seven significantly up-regulated and down-regulated circRNAs by microarray analysis

CircRNA	Type	Fold change	FDR	GeneSymble		P-value
**Up-regulated (HG vs NG)**						
hsa_circRNA_012448	exonic	66.115126	0.710323181		EPS15	0.0084424
hsa_circRNA_102129	exonic	47.0183016	0.710323181		MBTD1	0.0163760
hsa_circRNA_102230	exonic	35.374412	0.710323181		HGS	0.0080040
hsa_circRNA_103933	exonic	28.2309949	0.710323181		ADAMTS	0.0259363
**Down-regulated (HG vs NG)**						
hsa_circRNA_001586	sense overlapping	3.9494284	0.710323181		HIST1H3D	0.0003379
hsa_circRNA_400025	intronic	3.3494788	0.710323181		INCENP	0.0101937
hsa_circRNA_092556	sense overlapping	2.1979864	0.710323181		HIST1H1C	0.0035560

### Establishment of the circRNA–miRNA–mRNA network

**Figure 6.**
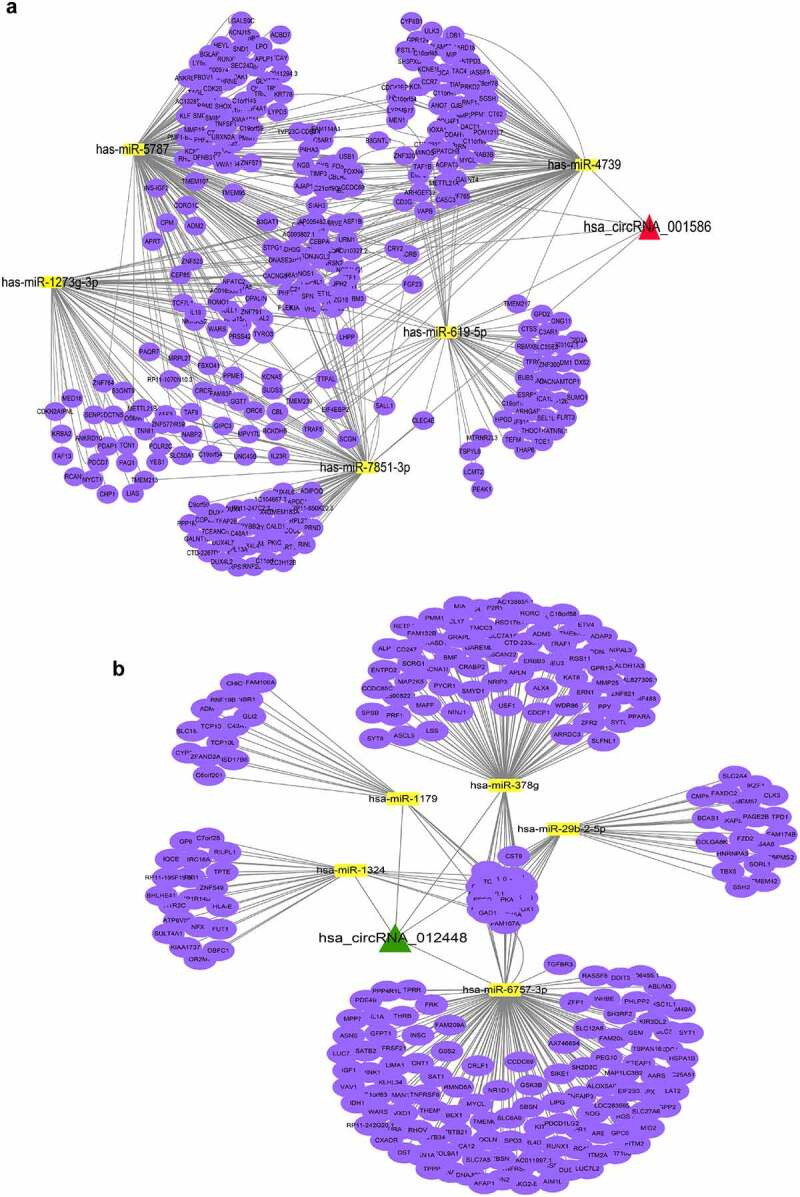
**Establishment of the circRNA–miRNA–mRNA network** (a) Establishment of the hsa_circRNA_001586-miRNA-mRNA network. (b) Establishment of the hsa_circRNA_012448-miRNA-mRNA network. The triangle nodes represent circRNAs. The rectangle nodes represent the hub miRNAs. The round nodes represent miRNAs target genes.

**Figure 7.**
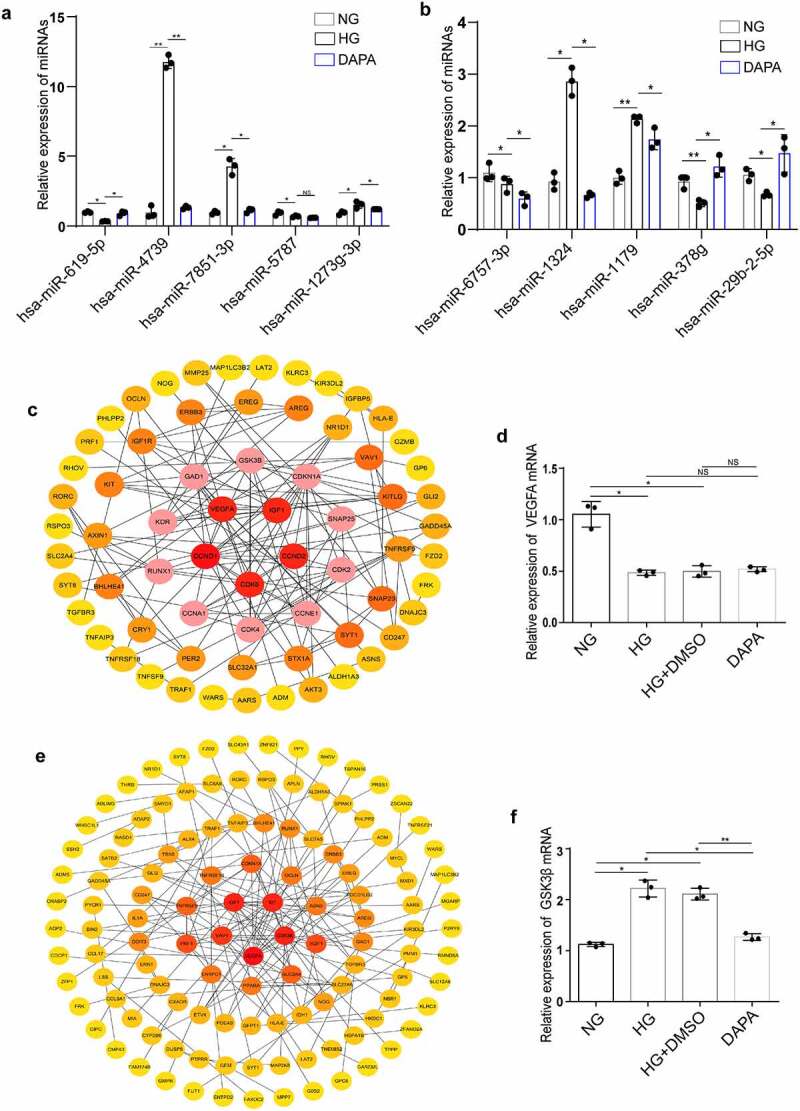
**Identification of the hub genes in the ceRNA networks involved in the protective effects of dapagliflozin on DKD** (a) Validation of the top five hub miRNAs associated with hsa_circRNA_001586 in the NG, HG, and dapagliflozin group by RT‐PCR. (b) Validation of the top five hub miRNAs associated with hsa_circRNA_012448 in the NG, HG, and dapagliflozin group by RT‐PCR. (c) The hub genes of the hsa_circRNA_001586-miRNA-mRNA network. (d) The expression of VEGFA in the NG, HG, and dapagliflozin groups by RT‐PCR. (e) The hub genes of the hsa_circRNA_012448-miRNA-mRNA network. (f) The expression of GSK3β in the NG, HG, and dapagliflozin groups by RT‐PCR. *P < 0.05, **P < 0.01, ***P < 0.001. NG: normal glucose, HG: high glucose, DAPA: dapagliflozin. Color shade represent the degree of correlation.

We further established the ceRNA networks of dapagliflozin-related circRNAs. We selected hsa_circRNA_001586, which was repressed by HG but induced by dapagliflozin, and hsa_circRNA_012448, which was induced by HG but repressed by dapagliflozin to share the common binding sites of the miRNA response element (MRE). In the hsa_circRNA_001586 related ceRNA network, the top five hub miRNAs including hsa-miR-619-5p, hsa-miR-4739, hsa-miR-7851-3p, hsa-miR-1273 g-3p, and hsa-miR-5787 were identified, and 336 miRNA target genes were obtained (Figure 6a). While in the hsa_circRNA_012448 related ceRNA network, the top five hub miRNAs including hsa-miR-1179, hsa-miR-378 g, hsa-miR-1324, hsa-miR-6757-3p, and hsa-miR-29b-2-5p were identified, and 262 miRNA target genes were obtained (Figure 6b). We then tested the expression of these miRNAs after dapagliflozin treatment, and found that hsa-miR-4739, hsa-miR-7851-3p, and hsa-miR-1273 g-3p were induced by HG but repressed by dapagliflozin (Figure 7a), whereas hsa-miR-378 g and hsa-miR-29b-2-5p were repressed by HG but induced by dapagliflozin (Figure 7b), and negatively correlated with the expression of hsa_circRNA_001586 and hsa_circRNA_012448, respectively. Based on the negative regulation between circRNAs and miRNAs, we speculate that hsa_circRNA_001586-miR-4739/miR-7851-3p/miR-1273 g-3p-mRNAs and hsa_circRNA_012448-miR-378 g/miR-29b-2-5p-mRNAs networks might participate in the protective effects of dapagliflozin on DKD.

## Identification of the hub genes in the ceRNA networks involved in the protective effects of dapagliflozin on DKD

To further explore the functions of the hsa_circRNA_001586 and hsa_circRNA_012448 related ceRNA networks, GO and KEGG analysis were performed. The results showed that gene expression, RNA metabolic process, amino acid metabolism, primary bile acid biosynthesis, and SNARE interactions in vesicular transport were enriched in the hsa_circRNA_001586-miR-4739/miR-7851-3p/miR-1273 g-3p-mRNAs network (Fig. S3a and b), whereas regulation of nitrogen compound metabolism, gene expression, MAPK, ErbB, transcriptional misregulation in cancer, and insulin resistance were enriched in the has_circRNA_012448-miR-378 g/miR-29b-2-5p-mRNAs network (Fig. S3c and d). Furthermore, we constructed PPI networks of the target genes involved in the ceRNA networks using the STRING database (http://string-db.org/). We showed the significant interactions between 67 hub genes in the hsa_circRNA_001586 related ceRNA network and found that VEGFA was one of the top five hub genes (Figure 7c). As a member of the PDGF/VEGF growth factor family, VEGFA was reported to protect the glomerular microvasculature in diabetes [39]. The results of RT-PCR further confirmed that the expression of VEGFA was downregulated in the HG group. However, dapagliflozin has no effect on the expression of VEGFA (Figure 7d). Meanwhile, we constructed the PPI network of hub genes in the hsa_circRNA_012448 related ceRNA network (Figure 7e) and found that GSK3β, which was reported to participate in the renal tubular injuries in DKD [40], was one of the top five hub genes. The results of RT-PCR showed that GSK3β was upregulated in the HG group but was downregulated in the dapagliflozin group (Figure 7f). After a series of analyses, the hsa_circRNA_012448-miR-378 g/miR-29b-2-5p-GSK3β network, which is likely to participate in anti-DKD mechanisms through direct and indirect effects, was eventually confirmed to be involved in the action of dapagliflozin against DKD.

## Hsa_circRNA_012448-hsa-miR-29b-2-5p-GSK3β was identified as an important pathway mediating the action of dapagliflozin against DKD

**Figure 8.**
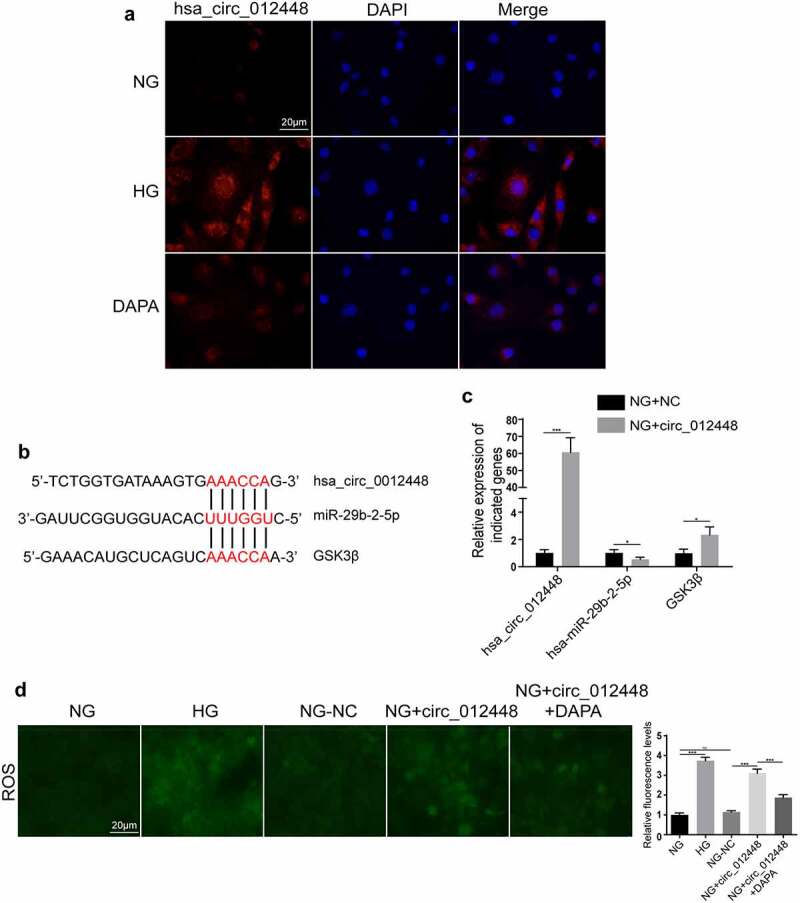
**Hsa_circRNA_012448-hsa-miR-29b-2-5p-GSK3β was identified as an important pathway mediating the action of dapagliflozin against DKD** (a) Distribution of hsa_circRNA_012448 in HK-2 cells by FISH assay. Scal bar: 20 μm. (b) The predicted binding sites between hsa_circRNA_012448, hsa-miR-29b-2-5p and GSK3β. (c) The expression of hsa_circRNA_012448, hsa-miR-29b-2-5p, and GSK3β in the hsa_circRNA_012448 overexpressing HK-2 cells. (d) Overexpression of hsa_circRNA_012448 increased the production of ROS in HK-2 cells, whereas dapagliflozin inhibited the generation of ROS. Scal bar: 20 μm. *P < 0.05, **P < 0.01, ***P < 0.001, NS: not significant. NC: normal controls; DKD: diabetic kidney disease. FISH: fluorescence in situ hybridization; ROS: reactive oxygen species; NG: normal glucose, HG: high glucose, DAPA: dapagliflozin.

Considering that the highest expression of hsa_circRNA_012448 was found in HK-2 cells induced by HG, we selected hsa_circRNA_012448 for further study. The FISH assay showed that hsa_circRNA_012448 was mainly distributed in the cytoplasm and that dapagliflozin reduced its expression in HK-2 cells induced by HG (Figure 8a), suggesting that dapagliflozin may regulate hsa_circRNA_012448 in HK-2 cell. Then, we predicated the interactions between hsa_circ_012448, hsa-miR-29b-2-5p, and GSK3β by performing bioinformatics analysis (Figure 8b). Surprisingly, we found that overexpression of hsa_circRNA_012448 reduced the expression of hsa-miR-29b-2-5p, but increased the expression of GSK3β in HK-2 cells (Figure 8c). Considering that GSK3β is an important regulator of the oxidative stress response, we further explored if hsa_circRNA_012448 participated in the regulation of the oxidative stress response in HK-2 cells. The results showed that overexpression of hsa_circRNA_012448 increased the generation of reactive oxygen species (ROS), which was reduced by dapagliflozin (Figure 8d). Collectively, these data indicated that hsa_circRNA_012448-hsa-miR-29b-2-5p-GSK3β might participate in the action of dapagliflozin against DKD, which is worthy of further investigation in the future.

## Discussion

Here, we used RNA microarray to detect the differential expression profiles of circRNA/mRNA in HK-2 cells treated with NG, HG, and different drugs (dapagliflozin, metformin, and vildagliptin), and explored the biological functions of circRNA/mRNA using network pharmacology and transcriptomics analyses. Furthermore, through the CNC networks, ceRNA networks, RT-PCR, and functional analysis, we identified hsa_circRNA_012448-hsa-miR-29b-2-5p-GSK3β as a potential signaling pathway mediating the protective effects of dapagliflozin on DKD.

Previous studies suggested that SGLT2i treatment exerts protective effects on DKD by regulating the tubuloglomerular feedback and reducing the blood glucose level [41]. However, the CREDENCE [10] and DAPA-CKD [11] studies have confirmed that DKD and non-DKD patients with renal dysfunction can benefit from SGLT2i treatment, suggesting that SGLT2i may protect renal function in a glucose-independent way. Furthermore, previous studies on the renal protection in patients with DKD conferred by metformin [42] and vildagliptin [43] focused on the whole renal function and hypoglycemic effect, while few studies investigated specific kidney components in DKD, such as renal tubular epithelial cells. Here, we identified a large number of DEcircRNAs, indicating that circRNAs play an important role in HK-2 cells treated with different drugs. Strikingly, although the network pharmacology predicted that the biological processes of dapagliflozin and vildagliptin on DKD were the lipid metabolism and insulin secretion signaling pathways, the parental genes of DEcircRNAs showed that dapagliflozin biological processes were involved in the Rap1 and transcription regulation in endoplasmic reticulum pathways, while those for vildagliptin were mainly involved in the HIF-1 and VEGF signaling pathways. However, the main pathways for metformin were the FOXO and AMPK signaling pathways, consistent with the results of a previous study [44]. These results indicated that the effects of dapagliflozin and vildagliptin may have tissue and cell specificity, extending the molecular mechanisms involved in the pathophysiological process of HK-2 cells.

Generally, a multi-drug combination approach is used for the treatment of diabetes and its complications. A previous study reported that the combination treatment of SGLT2i (dapagliflozin) and DPP-4i (saxagliptin) reduced the activation of the NLRP3 inflammasome and delayed DKD progression in mice with type 2 diabetes [45]. Furthermore, a new report indicated that metformin promoted autophagy in the kidneys of individuals with chronic kidney disease, possibly by contributing to the actions of SGLT2i in increasing autophagy and muting inflammation in diabetic kidneys [46]. Here, we performed functional analysis of circRNAs and mRNAs co-regulated by the three types of drugs. The results revealed several important pathways such as cell communication, cell death, transcription factor, MAPK, p53, ErbB, and HIF-1 signaling pathways. In addition, we identified the hub genes co-regulated by the three types of drugs. For example, VAV1 [47] and CDKN1A [48] are involved in cell proliferation, differentiation and cell cycle regulation. VEGFA plays a key role in the progression of renal fibrosis [49]. DDIT3 is involved in oxidative stress responses [50]. These findings suggested that the combined use of the three types of drugs may improve renal tubular injury by regulating PTEC proliferation, apoptosis, and fibrosis, providing guidance for further exploration of the treatment mechanisms of multi-drug combination *in vivo*.

Studies have indicated that SGLT2i are involved in renal tubular cell apoptosis, tubulointerstitial inflammation, and energy metabolism in DKD [51,52]. Based on the CNC networks, we revealed that the target genes of circRNAs in the HG group were associated with calcium ion transport, cell apoptosis, intercellular junctions, oxoacid metabolic process, amino acid metabolism, and lysosome pathways. However, these target genes of circRNAs in the dapagliflozin group were enriched in gene expression, glucose metabolic process, extracellular exosome, and amino acid metabolism pathways. Notably, extracellular exosome signaling provides us with a new possible mechanism. The exosomes secreted by renal tubules induced by dapagliflozin might affect the glomerulus, thereby improving glomerular function and restoring renal function. Collectively, these studies indicated that dapagliflozin exerts protective effects on DKD through multiple signaling pathways, some of which have not been previously studied.

CircRNAs could function as ceRNAs participating in the development of DKD [18,19]. Previous studies reported that hsa-miR-4739 is elevated in the urinary exosomes of patients with DKD [53]. Furthermore, hsa-miR-378 g not only participates in the fat metabolism in obese mice, but also alleviates renal tubulointerstitial fibrosis [54]. Our study showed that hsa_circRNA_001586 and hsa_circRNA_012448 could function as the ceRNAs of hsa-miR-4739 and hsa-miR-378 g, which were involved in the cell adhesion and junctions, amino acid metabolism, vesicle transport, MAPK, and insulin secretion signaling pathways. Using RT-PCR, we found that the level of hsa_circ_012448 was increased in cell culture in DKD and regulated hsa-miR-29b-2-5p-GSK3β signaling, indicating that hsa_circRNA_012448-hsa-miR-29b-2-5p-GSK3β may be a potential pathway for dapagliflozin in protecting DKD. Thus, these ceRNA networks may be new biomarkers and therapeutic targets for DKD. These mechanisms need to be further verified using *in vivo* and *in vitro* experiments as well as large-scale clinical trials in the future.

## Conclusion

In summary, this is the first study to construct the circRNA/mRNA expression profile of HK-2 cells treated with NG, HG, dapagliflozin, metformin, and vildagliptin. Through the circRNA-mRNA, circRNA-miRNA-mRNA networks, and functional analysis, we provided important insight into the regulatory functions and potential molecular mechanisms of dapagliflozin in the pathogenesis of DKD.

## Supplementary Material

Supplemental Material

## Data Availability

All raw data were deposited in the Gene Expression Omnibus under the accession code GSE179226 and GSE158534.

## Research Highlights

Dapagliflozin/metformin/dipeptidyl peptidase-4 inhibitor target circRNAs/mRNAs in PTECs of DKD;

Dapagliflozin may protect DKD through lipid metabolism, Rap1, and MAPK signaling pathways;

The hsa_circRNA_012448-hsa-miR-29b-2-5p-GSK3β pathway plays an important role in the action of dapagliflozin against DKD.
