# Supplementary material for: Identification of circular RNAs and functional competing endogenous RNA networks in human proximal tubular epithelial cells treated with sodium-glucose cotransporter 2 inhibitor dapagliflozin in diabetic kidney disease
Source: Bioengineered. 2022 Feb 7;13(2):3911–29. doi: 10.1080/21655979.2022.2031391 (PMC8973950; doi:10.1080/21655979.2022.2031391)
Supplement: Supplemental Material [file KBIE_A_2031391_SM3519.docx]

**Identification of circular RNAs and functional competing endogenous RNA networks in human proximal tubular epithelial cells treated with sodium-glucose cotransporter 2 inhibitor dapagliflozin in diabetic kidney disease**

Yi Song,^1,2^ Feng Guo,^1,2^ Yifan Liu,^3^ Fengjuan Huang,^2^ Xunjie Fan,^1,2^ Lin Zhao,^2^ Guijun Qin,^2,^*

*Correspondence: Guijun Qin

Department of Endocrinology and metabolism, the First Affiliated Hospital of Zhengzhou University, Zhengzhou 450052, China

Tel: +86-13598860123

Email: [hyqingj@zzu.edu.cn](mailto:hyqingj@zzu.edu.cn)

**Supplementary figures**

**Fig. S1**


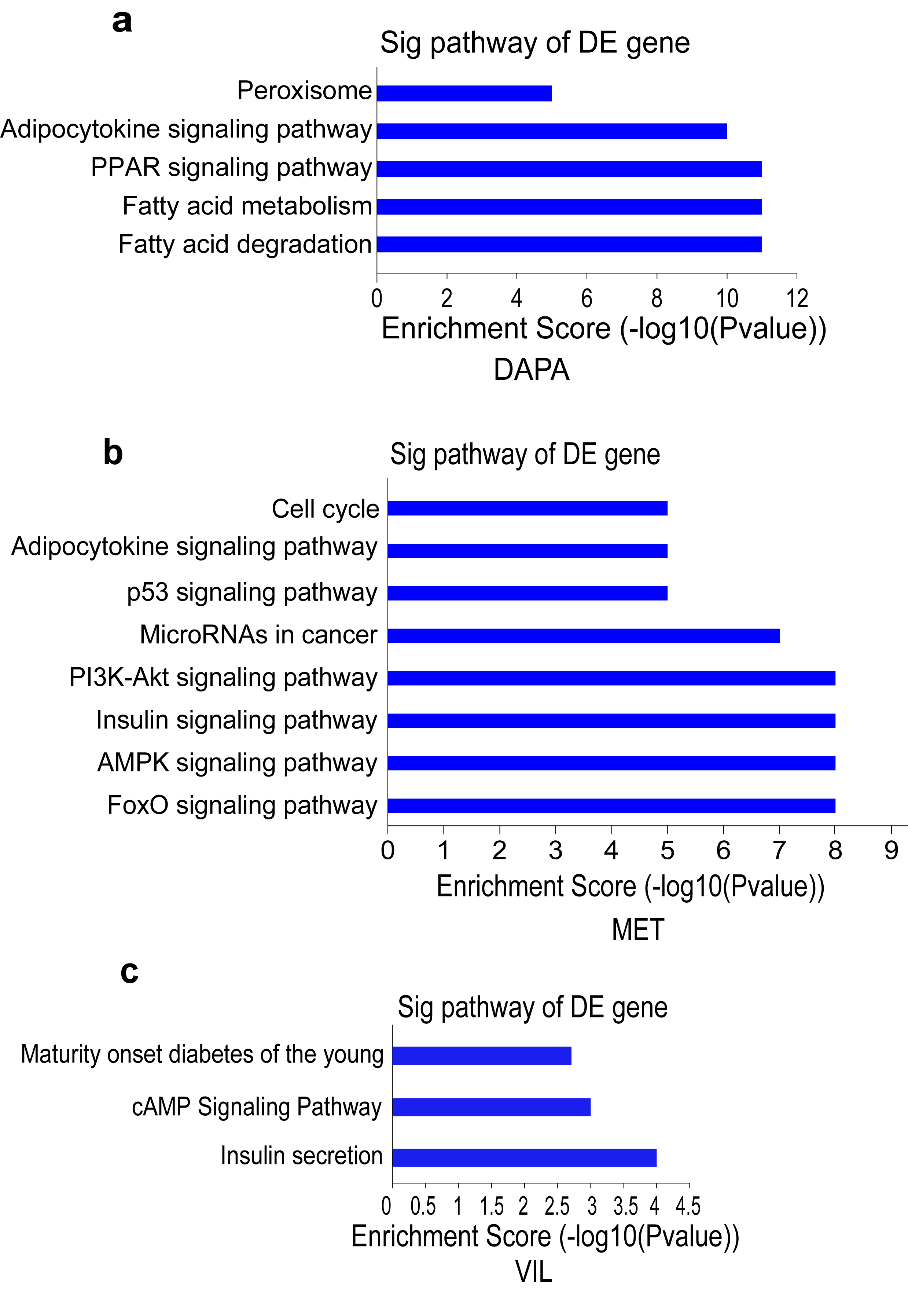


**Fig. S1** KEGG pathway analyses for the predicted target genes of different drugs (**a**. dapagliflozin, **b**. metformin, **c**. vildagliptin) on DKD using STITCH database.

**Fig. S2**


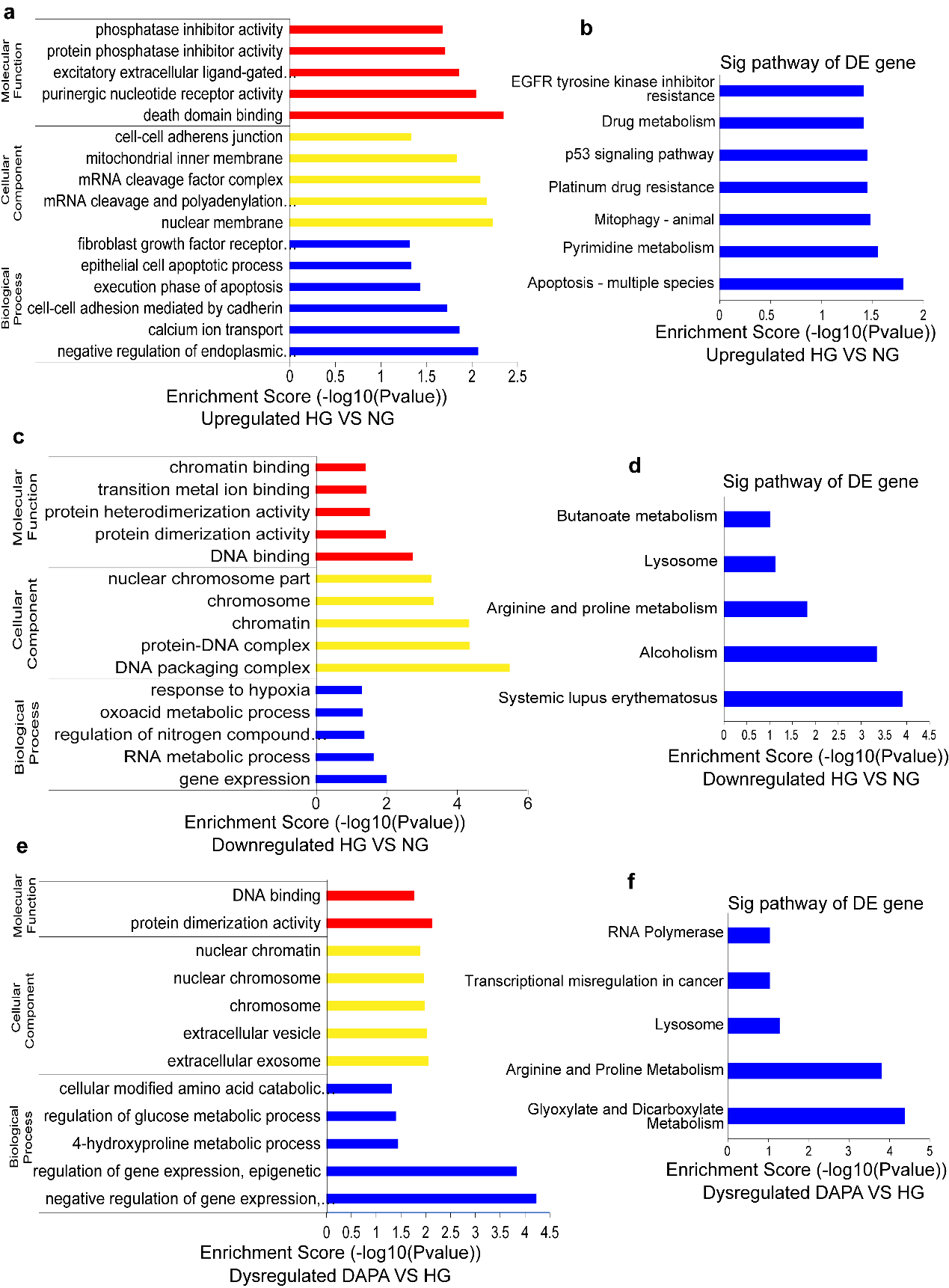


**Fig.S2** **Functional analyses for circRNA-mRNA interaction network.** (**a**)-(**b**) GO (**a**) and KEGG pathway (**b**) analyses for the up-regulated circRNAs in the HG group compared with the NG group. (**c**)-(**d**) GO (**c**) and KEGG pathway (**d**) analyses for the down-regulated circRNAs in the HG group compared with the NG group. (**e**)-(**f**) GO (**e**) and KEGG pathway (**f**) analyses for the dysregulated circRNAs in the dapagliflozin group compared with the HG group. NG: normal glucose, HG: high glucose, DAPA: dapagliflozin.

**Fig.S3**


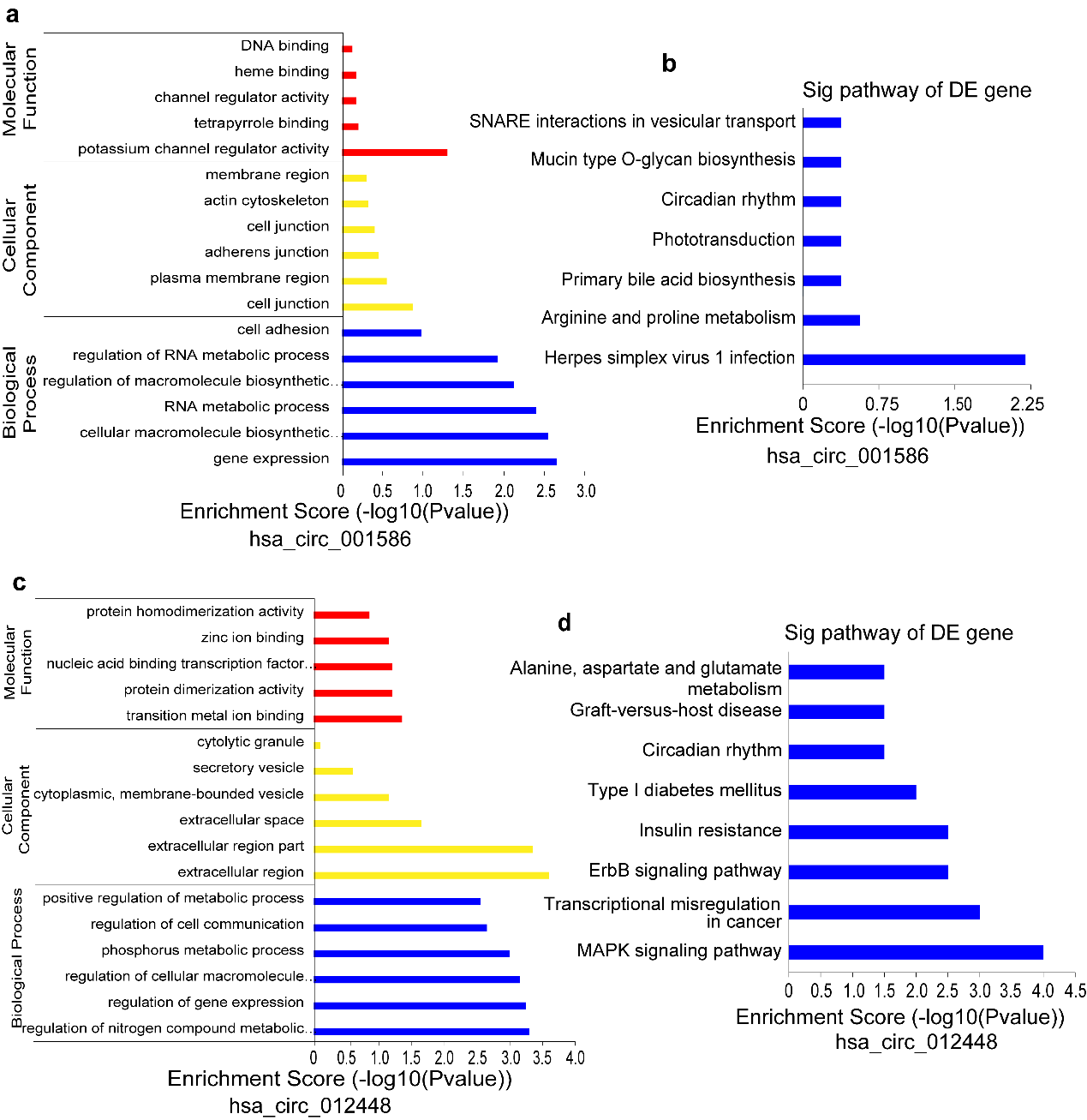


**Fig. S3** GO and KEGG pathway analyses for the target genes of circRNA-miRNA-mRNA networks. (**a**)-(**b**) GO (**a**) and KEGG pathway (**b**) analyses for the target genes of the hsa _circRNA_001586-miRNA-mRNA network. (**c**)-(**d**) GO (**c**) and KEGG pathway (**d**) analyses for the target genes of the hsa _circRNA_012448-miRNA-mRNA network.
